# A conversation with Jonathan M. Davis, MD, Vice-chair of Pediatrics, Tufts Medical Center, Tufts University School of Medicine

**DOI:** 10.1017/cts.2025.10046

**Published:** 2025-05-30

**Authors:** Clinical Research Forum

**Affiliations:** Clinical Research Forum, Washington, DC, USA

## Top 10 clinical research achievement awards Q & A

This article is part of a series of interviews with recipients of Clinical Research Forum’s Top 10 Clinical Research Achievement Awards. This interview is with Jonathan M. Davis, MD, Vice-Chair of Pediatrics, Tufts Medical Center. Dr Davis has served on many federal advisory panels and is currently Chair of the Neonatal Advisory Committee and an ad hoc member of the Pediatric Advisory Committee in the Office of Pediatric Therapeutics at the US Food and Drug Administration. His research focuses on neonatal abstinence syndrome (babies born addicted to opioids) and developing new therapeutics (drugs, biologics, devices) to improve neonatal outcomes. Dr Davis received a 2024 Top 10 Clinical Research Achievement Award for The GEMINI Study. *The interview has been edited for length and clarity.*

## When did you first become interested in clinical research?

My interest in clinical research started during my second year of medical school. I had a pharmacology professor who was a neonatologist, and during one of his lectures, he explained how premature babies are sometimes given caffeine to help prevent apnea of prematurity. That fascinated me, so I approached him afterwards and asked if I could work in his lab. He took me in, and I was able to work on multiple projects, even winning an award for outstanding student research. I went on to residency at Boston Children’s Hospital, where I worked with the prominent neonatologist and pediatrician Dr Mary Ellen Avery. After that, I was really hooked.

## How do you strike the balance between patient care and research?

For me, striking the balance starts with patient care – it’s what has fueled my whole career. I’ve been Chief of Newborn Medicine for 33 years, and every morning when I go into the neonatal intensive care unit (NICU) for rounds, I’m continually intrigued by how we can make things better for our patients. I’ve always felt that my research efforts need to be directly focused on improving outcomes, and that includes our research work in the NICU and laboratory, as well as advocacy to develop policies and procedures that help patients on a broader global scale.

## You have been very involved in advocacy for policy change to end prenatal opioid abuse and better treat neonatal abstinence syndrome. How did that start?

We were doing rounds in the NICU, and a Fellow pointed out two babies – both had mothers who took methadone for treatment of opioid use disorder during their pregnancies. One baby was much sicker with opioid withdrawal, and the other had mild signs of withdrawal. We started asking questions about why and what we could be doing better, so I called my former professor (the one who taught the pharmacology course), and he suggested looking at the genetics. We did, and when that research was published in JAMA, it opened up a whole new area of study around the genetics and epigenetics of opioid withdrawal. At the time, I was supervising care at Melrose Hospital in addition to my role at Tufts, and I got a call from U.S. Rep. Catherine Clark’s office. She’s now Minority Whip of the United States House of Representatives, but at the time, she was a freshman Congresswoman interested in the opioid epidemic. We started working together (along with Mitch McConnell in the Senate), and we worked together on the passage of the Protecting Our Infants Act of 2015, which aims to combat the rise of prenatal opioid abuse and neonatal abstinence syndrome, keeping families intact. That got me working on more efforts related to advocacy, first at a national level and then worldwide. Never in a million years did I think that I would be involved in this kind of advocacy, but it’s been extremely motivating and gratifying to use my medical and research training in this meaningful way.

## Tell us about the award-winning research

The Genomic Medicine in Ill Infants and Newborns (GEMINI) study has been incredible and is probably the most enjoyable research project I’ve ever worked on. Remember, I’m not a geneticist, but I have had a longstanding interest in exploring if there’s a genetic basis to why some NICU babies are so sick. This trial was about evaluating different genetic tests. We enrolled sick babies who underwent simultaneous testing with genomic sequencing and a commercially available, targeted neonatal gene-sequencing panel. Then we compared the results, looking at molecular diagnostic yield, time to diagnosis, and clinical utility.

## What were the findings?

The molecular diagnostic yield for genomic sequencing was higher than a targeted neonatal gene-sequencing test (49% vs 27%), but the time to return for routine results was slower (6.1 vs 4.2 days). GEMINI also identified 134 novel variants in genes directly related to phenotype; 73 potentially causal variants were classified as a VUS (variant of uncertain significance).

## How could these results change newborn care?

We found that genome sequencing was nearly twice as effective as targeted testing in picking up genetic disorders. That means, if the cost can get low enough and coverage can be expanded, replacing current newborn screening with genomic sequencing could dramatically improve care for newborn babies. It would be a paradigm shift in clinical practice. What’s interesting is that when you examine the economic modeling, if one moves right to genome sequencing, which eliminates 40 or 50 other genetic or metabolic tests that are often done first, there’s a savings of $155,000 per patient on average. One economist said that’s the largest cost savings of any testing ever studied in the last 50 years, for all of medicine, not just in neonatology.

## Where is this research headed next?

We want to be able to make a diagnosis of a genetic disorder much earlier, instead of waiting to see how these infants do after they leave the NICU. About half the patients in our study were not diagnosed with a disorder, and yet they were so sick. We’re now going back and re-analyzing the data – because more data has been added to genetic databases and some patients are now exhibiting traits that weren’t identifiable in the NICU. Of the first 29 patients that we have re-analyzed, eight now have a diagnosis. Of course, expanding the opportunity to make a diagnosis is only part of the story. We also need to help these kids go forward with treatments, if possible – before there are more complications and problems. Since we can identify genetic disorders earlier and earlier, there’s an opportunity for an explosion of gene and cell-based treatments for rare diseases in newborns.

## Are some kids already benefiting from whole genome sequencing?

Yes, it’s already making a huge difference for some patients. We had one baby in the trial who had been in the NICU for months with an undiagnosed life-threatening condition. We ran the genome sequencing and found out that she had a very rare bleeding disorder and another condition, malignant hyperthermia, which causes a severe reaction to certain anesthesia drugs. Remarkably, those results came when she was on her way to surgery for a brain bleed, and that procedure was stopped until a different anesthesia plan was developed. She’s now four years old and has a normal life, with the help of periodic infusions of a clotting agent. This is just one example of many powerful stories that are so inspiring to researchers and other folks on the advocacy side of things.

## What advice would you give those who are just beginning their careers in clinical research?

What I like to tell young people is this: Don’t think for a second that one person can’t make a difference. Sure, at first, the challenges may seem too big or too striking, but just start small. Look at a particular clinical question and determine where you have the passion, the drive, and the interest to have an impact. Why wait to read about it in a journal or have someone else tell you what to do? It’s so much more fun to find the answers yourself, to do the research, and then translate your findings to make a difference in people’s lives. When I was starting my career, I knew absolutely nothing about opioids, outside of how to treat a baby with opioid dependence. I kept doing the work and learning and now, I’m advising the office of the Assistant Secretary of Health about programs that address the opioid epidemic and pregnant women. The same is happening with GEMINI. We started out small, but we will continue to evolve as the research does. It’s great to be part of the process and know that you are having a positive impact.

